# Supplementary figures and images for: CaBagE: A Cas9-based Background Elimination strategy for targeted, long-read DNA sequencing
Source: PLoS One. 2021 Apr 8;16(4):e0241253. doi: 10.1371/journal.pone.0241253 (PMC8031414; doi:10.1371/journal.pone.0241253)

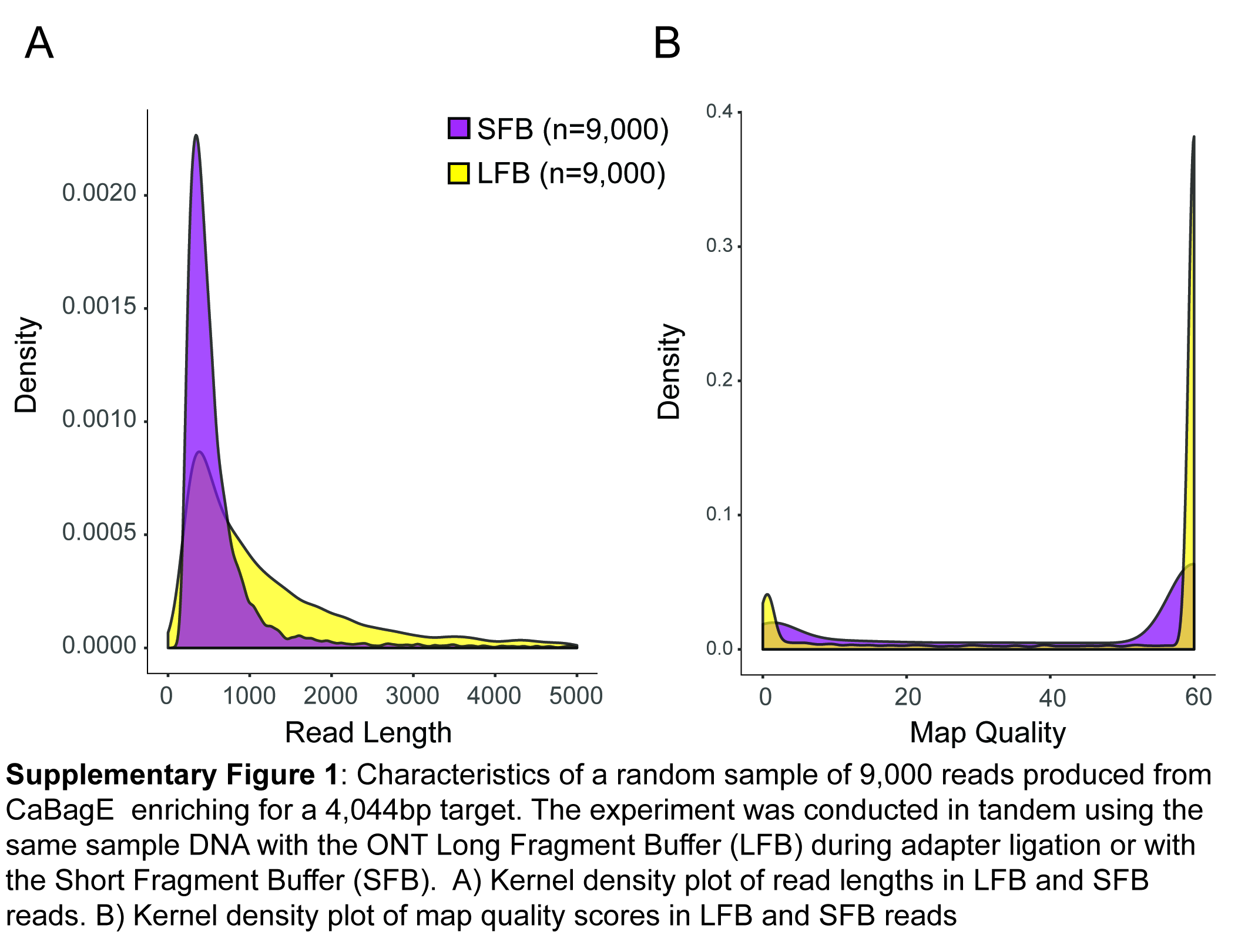

Supplement: S1 Fig — Characteristics of a random sample of 9000 reads produced from a CaBagE run enriching for a 4,044bp target. The experiment was conducted in tandem using the same sample DNA with the ONT Long Fragment Buffer (LFB) during adapter ligation or with the Short Fragment Buffer (SFB). A) Kernel density plot of read lengths in LFB and SFB reads. B) Kernel Density plot of map quality scores in LFB and SFB reads. (TIF) [file pone.0241253.s001.tif]

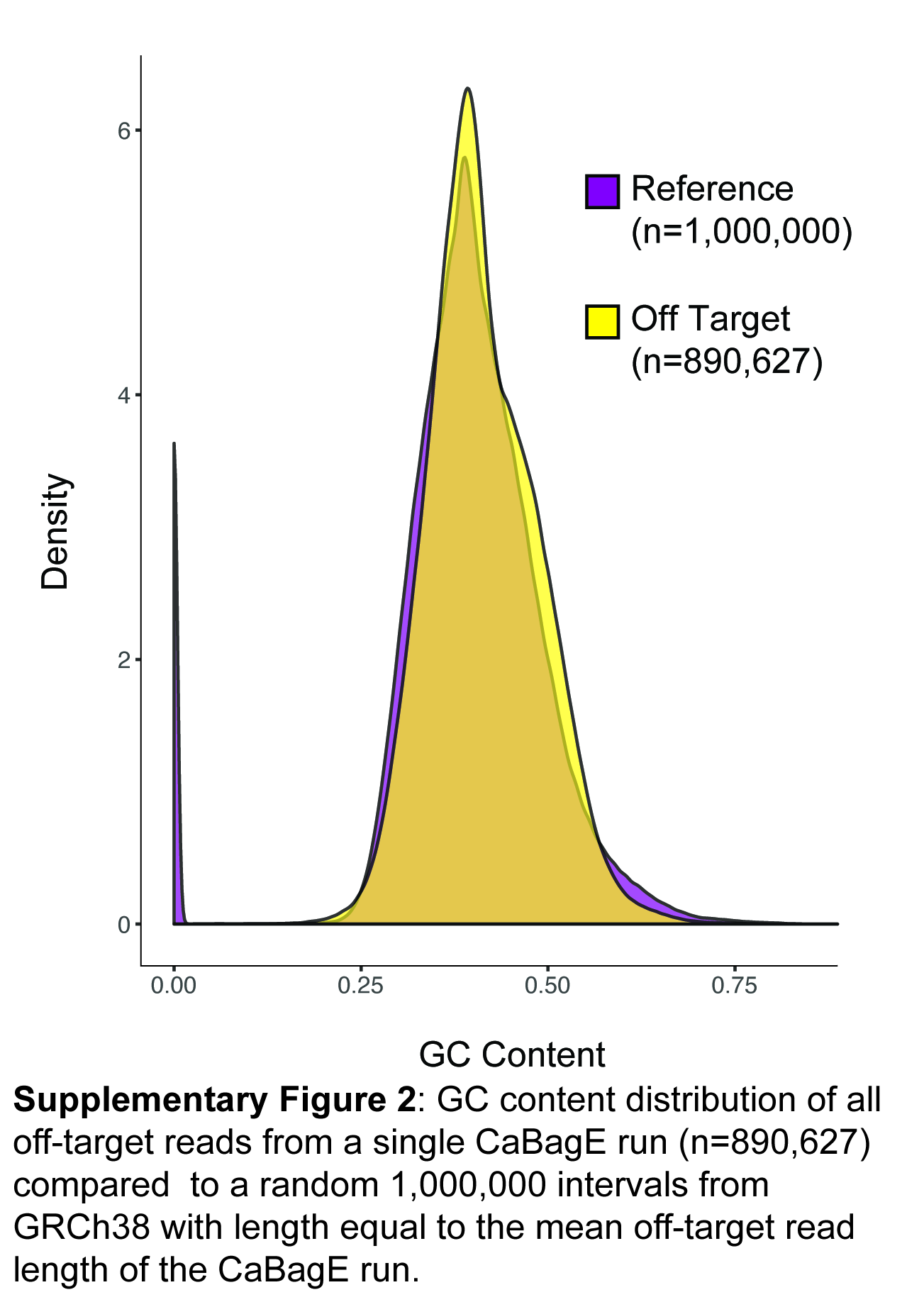

Supplement: S2 Fig — GC content distribution of all off-target reads from a single CaBagE run (n = 890,627) compared to a random 1,000,000 intervals from GRCh38 with length equal to the mean off-target read length of the CaBagE run. (TIF) [file pone.0241253.s002.tif]

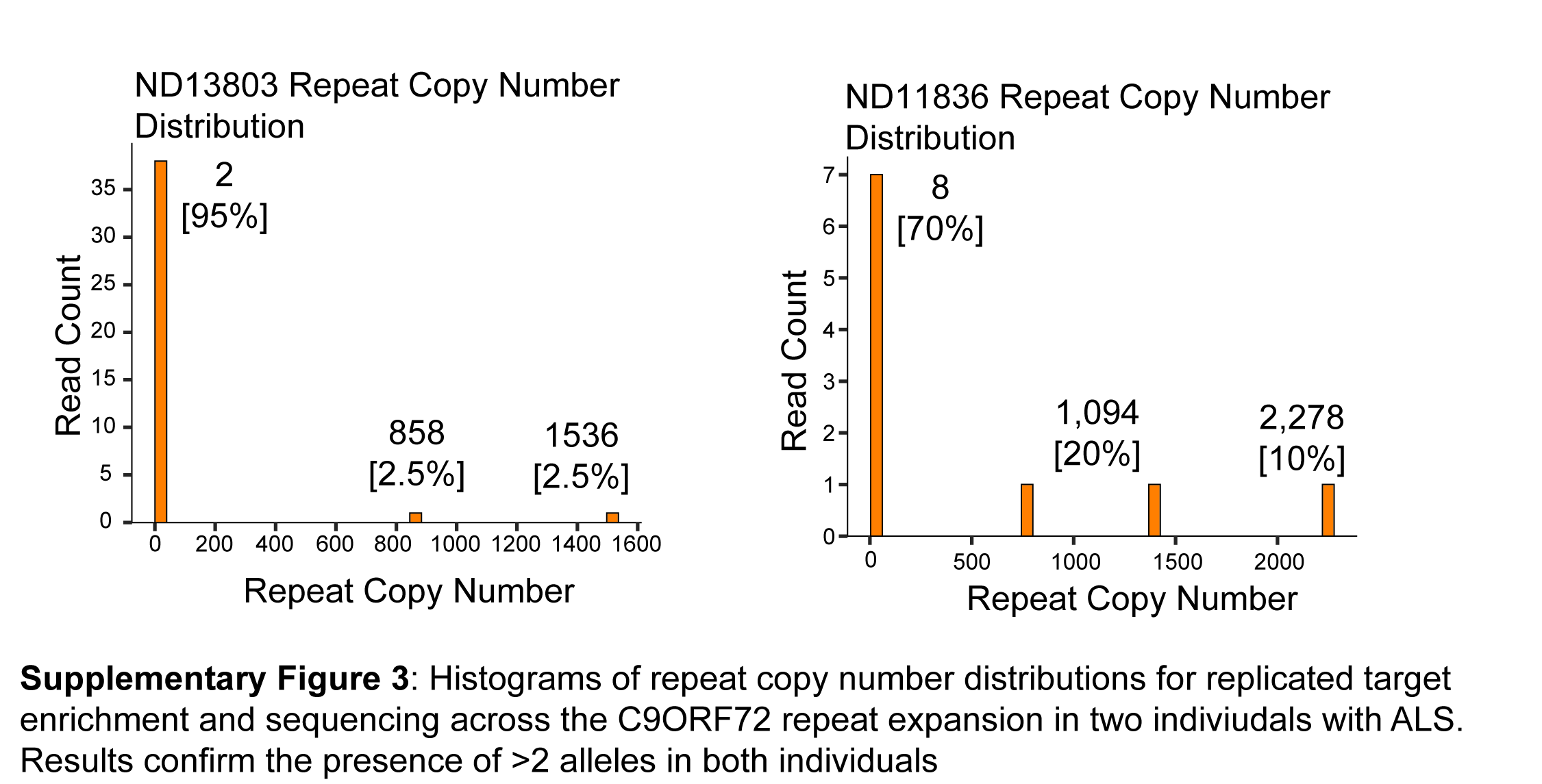

Supplement: S3 Fig — Histograms of repeat copy number distributions for replicated target enrichment and sequencing across C9orf72 repeat expansions in two individuals with ALS. Results confirm presence of >2 alleles in both individuals. (TIF) [file pone.0241253.s003.tif]
