# Supplementary material for: CaBagE: A Cas9-based Background Elimination strategy for targeted, long-read DNA sequencing
Source: PLoS One. 2021 Apr 8;16(4):e0241253. doi: 10.1371/journal.pone.0241253 (PMC8031414; doi:10.1371/journal.pone.0241253)
Supplement: S1 Raw images — (PDF) [file pone.0241253.s006.pdf]

Fig 1. Raw image

Filename: 16270x1-12_SampleQC.HSD1000

Gel Image

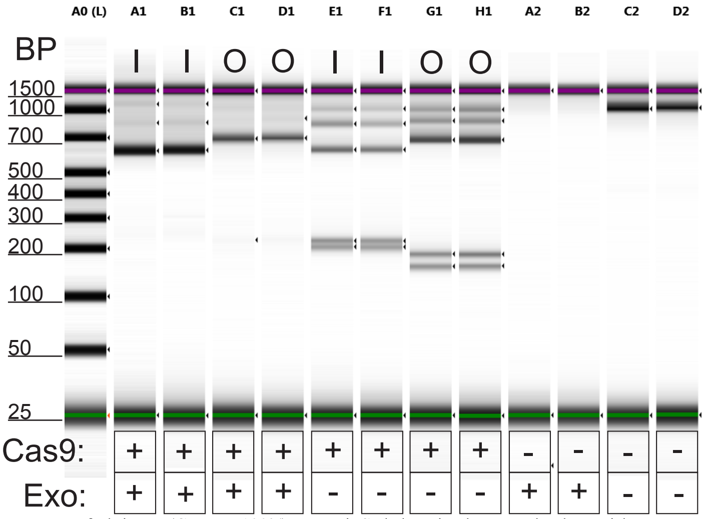

Default image (Contrast 100%), Image is Scaled to view larger Molecular Weight range

Sample Info

Well	Conc. [pg/µl]	Sample Description	Alert	Observations
A0	2350	Electronic Ladder		Ladder
A1	299	16270x1		
B1	284	16270x2		
C1	114	16270x3		
D1	122	16270x4		
E1	264	16270x5		
F1	233	16270x6		
G1	353	16270x7		
H1	389	16270x8		
A2	0.902	16270x9		
B2		16270x10		
C2	227	16270x11		
D2	192	16270x12		
