# Supplementary material for: CaBagE: A Cas9-based Background Elimination strategy for targeted, long-read DNA sequencing
Source: PLoS One. 2021 Apr 8;16(4):e0241253. doi: 10.1371/journal.pone.0241253 (PMC8031414; doi:10.1371/journal.pone.0241253)
Supplement: Attachment — Submitted filename: Review_manuscript_CaBaGe.docx [file pone.0241253.s007.docx]

**CaBagE: a Cas9-based Background Elimination strategy for targeted, long-read DNA sequencing**

This manuscript explains a modified version of a recent methodology named nCats or simply Cas9-enrichment (Gilpatrick et al., 2020 and López-Girona et al., 2020) that is being used in human, bacteria and just recently in plants, to enrich targeted long genomic loci (that might be challenging to capture by short read sequencing approaches due to complex haplotype structures such as those coming from tandem repeats) and get them sequenced at high coverage by using the long read Oxford Nanopore sequencing technology (ONT). The differences of CaBagE versus nCats are two steps in the library preparation. The nCast methodology produces dephosphorisation of the total genomic DNA before Cas9 cleavage, which it exposes ligatable ends to get targeted attachment of adaptors to the region of interest. Thus, nCast enrichment happens at the sequencing level, because both the target DNA and the rest of the genome remain in the library and are loaded into the MiniON flowcell, but just the target gets sequenced (plus some off-targets or pieces that get adapters due to not complete de-phosphorylation). CaBagE doesn’t perform de-phosphorylation, it does also Cas9 digestion and then this is followed by a long step (2 hours at 37C digestion plus 20min at 80C deactivation) of exonuclease digestion to remove any DNA but the targeted loci that will be protected at both flanking sides by Cas9-RNP complexes. Both methods use the same MiniON ligation kit and flowcells for sequencing. Therefore, CaBagE’s enrichment happens at the DNA level by removing non-target DNA during the exonuclease digestion step, so that ideally just the target loci will be loaded and sequenced by ONT pores or at least that the ratio on-to off-target DNA will increase before sequencing.

**Some aspects that need some improvement from my point of view:**

In the introduction is mentioned that many emerging methods using the specific targeting ability of the CRIPSR/Cas9 system are being developed but then you just cite 3 of them. I have found these references that might be nice to cite here as well: (López-Girona et al., 2020; Watson et al., 2020) and also (Bennett-Baker & Mueller, 2017; Jiang et al., 2015; Jiang & Zhu, 2016; Lee, Larionov, & Kouprina, 2015; Loose, 2018).

**Figure 2A**. It would be nice to add the orientation of the guide RNAs followed by their PAMs at either side of the scissors somehow (maybe a simple arrow will do the job), so it would be clearer what PAM-in and PAM-out orientation means by simply looking the figure.

Very nice and smart thing to compare CaBagE and nCATs side by side using the same 4 loci used by Gilpatrick et al., 2020, so you already knew about the good performance of guide RNAs to those targets. Although, at the end CaBagE yielded less targeted coverage than nCATs after this test. I agree with what you mentioned in line 238 that this might be due to an incomplete exonuclease digestion. However, the discussion about the performance of CaBagE versus nCats in lines 287-293 is a bit vague. I understand that you want to highlight the good qualities of your method, but I think this paper will be more cited if you also comment on the applicability of your method versus nCat. Why people should try CaBagE if yields less coverage and implies an added long 2h 20min step (added cost with several exonucleases versus alkaline phosphatase for de-phosphorylation)? You mentioned that might be useful to increase specificity during size separation or for PCR-free cloning.

CRISPR-Cas9 enrichment approaches that don’t imply an exonuclease digestion after Cas9 cleavage might still advantageous against your method due to the fact some input DNA molecules might be already fragmented due to normal DNA shearing during DNA extraction. These DNA molecules will have just one of the two crispr sites and therefore will be specifically digested by Cas9 but they will be removed or digested from your library during the exonuclease step due to the fact that they won’t be protected in one of the two flaking regions. Or in other words, exonucleases will be removing from your library DNA molecules that could still be sequenced from one side (the side containing the crispr site and protected by Cas9 that will end that having an ONT adaptor and therefore being sequenced though the pore). Thus, these single side sequences will be still useful for increasing the overall coverage of your targets even if they don’t cover the whole region but they might be long enough to decipher the copy number repeat. But by CaBagE you restrict the coverage to those un-fragmented and perfectly Cas9-digested DNA molecules.

By removing the single side sequencing reads you also restrict the use of the CRISPR-Cas9 enrichment methodology to other further applications. An example, can be those cases where you want to target a long loci but you only know one of the flanking regions of it. So, you could perform a genome walking approach to discovery the unknown region but with CaBagE you would destroy all those DNA molecules by the exonuclease reaction. It might also restrict it uses in determining haplotype dosage in polyploid species; not a problem for human’s targets however might help when trying to retrieve the whole representation of haplotypes in cases where somatic mosaicism happens. Because, as you said there is a strong bias toward shorter reads and CaBagE introduces another bias towards DNA molecules Cas9-digested at both sides, this might end up hiding some interesting haplotypes that they simply won’t get sequenced. So, again I think it would be nice if you argument in discursion the applicability of this method against the CRISPR-Cas9 enrichment explained at (Gilpatrick et al., 2020 in humans and in López-Girona et al., 2020 in plants).

An interesting point to highlight at discussion section would be to add a **table including price and time comparison between doing nCats and CaBagE.** This should be easy giving the fact that you are using 4 of the same gene targets that nCats authors used. Including the cost of enzymes, and times of each step. So, you could fight for the use of CaBagE from the labour cost side perspective although nCats might be better choice for obtained higher coverage.

The manuscript is well written and structured. However, the section **‘repeat copy number estimation’ on discussion around line 386** needs some attention. The long paragraph starting by ‘Next, spanning reads were realigned…’ needs to be broken in different sentences for clarification or be explained in a simpler way as it gets confusing at the second line.

**Methods:** Why do you use sequencing buffer (SQB) with water and flushing buffer (FB) for priming the pore rather than using FB with sequencing tether? This is a modification from the usual protocol. Do you see any improvement in the library run by doing this? This will be nice to comment in the discussion as it will be useful for other ONT sequencing approaches and many ONT users will be kind on knowing.

**Availability of data and code:** You should provide a Github repository link (or similar service, bitbucket, source forge, gitlab…) containing the analysis code used in this study as well as provide access to the raw and sequencing data generated.

Overall very nice manuscripts that after these improvements will be highly accessed by the research community that is improving and advancing on the *in vitro* CRISPR-Cas9 enrichment methodology in different species.

Bennett-Baker, P. E., & Mueller, J. L. (2017). CRISPR-mediated isolation of specific megabase segments of genomic DNA. *Nucleic Acids Research*, *45*(19), e165. https://doi.org/10.1093/nar/gkx749

Gilpatrick, T., Lee, I., Graham, J. E., Raimondeau, E., Bowen, R., Heron, A., … Timp, W. (2020). Targeted nanopore sequencing with Cas9-guided adapter ligation. *Nature Biotechnology*, *38*(4), 433–438. https://doi.org/10.1038/s41587-020-0407-5

Jiang, W., Zhao, X., Gabrieli, T., Lou, C., Ebenstein, Y., & Zhu, T. F. (2015). Cas9-Assisted Targeting of CHromosome cloning of large gene clusters. *Nature Communications*, *6*, 1–8. https://doi.org/10.1038/ncomms9101

Jiang, W., & Zhu, T. F. (2016). Targeted isolation and cloning of 100-kb microbial genomic sequences by Cas9-assisted targeting of chromosome segments. *Nature Protocols*, *11*(5), 960–975. https://doi.org/10.1038/nprot.2016.055

Lee, N. C. O., Larionov, V., & Kouprina, N. (2015). Highly efficient CRISPR/Cas9-mediated TAR cloning of genes and chromosomal loci from complex genomes in yeast. *Nucleic Acids Research*, *43*(8), e55–e55. https://doi.org/10.1093/nar/gkv112

Loose, M. (2018). Finding the Needle: Targeted Nanopore Sequencing and CRISPR-Cas9. *The CRISPR Journal*, *1*(4), 265–267. https://doi.org/10.1089/crispr.2018.29028.mlo

López-Girona, E., Davy, M. W., Albert, N. W., Hilario, E., Smart, M. E. M., Kirk, C., … Chagné, D. (2020). CRISPR-Cas9 enrichment and long read sequencing for fine mapping in plants. *Plant Methods*, *16*, 121. https://doi.org/10.1186/s13007-020-00661-x

Watson, C. M., Crinnion, L. A., Hewitt, S., Bates, J., Robinson, R., Carr, I. M., … Bonthron, D. T. (2020). Cas9-based enrichment and single-molecule sequencing for precise characterization of genomic duplications. *Laboratory Investigation; a Journal of Technical Methods and Pathology*, *100*(1), 135–146. https://doi.org/10.1038/s41374-019-0283-0
