# Supplementary material for: CaBagE: A Cas9-based Background Elimination strategy for targeted, long-read DNA sequencing
Source: PLoS One. 2021 Apr 8;16(4):e0241253. doi: 10.1371/journal.pone.0241253 (PMC8031414; doi:10.1371/journal.pone.0241253)
Supplement: Attachment — Submitted filename: Cabage_PLOS_Response_to_Reviewers_v4.docx [file pone.0241253.s008.docx]

We thank the reviewers for their insightful comments. We have incorporated the vast majority of suggestions, and feel that the manuscript is greatly improved, particularly regarding relevant literature citations and areas needing clarification. Below are our responses to each comment made by the reviewers. The original reviewer comments (italics) are followed by our response and relevant changes to the manuscript (bold).

Reviewer #1

1. *The enrichment strategy described in this paper … has already been described elsewhere … and termed Negative Enrichment. The author should at least cite the original method in the Introduction section and highlight similarities/differences with their CaBagE strategy, if any (otherwise, the novelty claim should be toned-down as well).*

We thank the first reviewer for bringing this manuscript to our attention. The reviewer is correct that the Negative Enrichment (NE) strategy is conceptually the same as CaBagE and so we have removed all statements of novelty regarding CaBagE from the manuscript and have cited NE in the introduction (**Line 144**). After sequencing with ONT, the NE strategy yielded less than 15 on-target reads. There are differences between the NE and CaBagE strategies that result in the 3- to 32-fold higher on-target sequence depth with CaBagE after long read sequencing. These differences include the amount of DNA input, choice of exonucleases, length of exonuclease digestion, and library preparation protocol. Further, the authors of the NE protocol do not specify how or whether the exonucleases were inactivated or Cas9 was dissociated from the target; or the orientation of the guide RNAs (PAM orientation). The differences likely contributing to the improved performance of CaBagE over NE are reviewed in the discussion (**Line 325**).

2. *…Median (or mean) read depth should be stated clearly in the Abstract in order not to create inflated expectations.*

This point is well taken. The abstract now states the median (and range) read depth achieved by CaBagE, rather than the maximum achieved read depth (**Line 65**).

3. *… Can the authors elaborate on the number of reads overlapping a larger portion of the target region (for instance, 50% of the region of interest)? It would be nice to have a column specifying such a value for each sequencing run/region of interest in Table 1…*

As per the reviewer’s recommendation, we have added a column to **Table 1** that shows the count of reads that overlap the region of interest by 90% or more (termed ‘spanning reads’). As shown in the updated Table, most on-target reads are also spanning reads and the proportion (59-97%) appears to correlate with target size. These new results are discussed on **Lines 225-230.**

4. *Which minimap2 parameters do the authors use to align sequenced reads? In principle, the standard presets for Nanopore Sequencing data (-x map-ont) are not expected to give the best alignment results...*

We have added the Minimap2 parameters used to the methods section on **Line 451.** We had two concerns regarding capturing reads with large repeat expansions. The first was that base calling might fail, and therefore expanded reads would not be aligned. We accounted for this by aligning reads that failed to meet default quality thresholds for base calling with guppy, in addition to the reads that passed, which allowed us to recover a small number of additional on-target reads. This is discussed in the methods (**Line 473)** and in the discussion (**Line 380**). The second concern, as mentioned by the reviewer, was that we might fail to identify reads that span the *C9orf72* repeat expansion in carriers due to clipping by Minimap2. We took several steps to mitigate this possibility. The first was to design the guideRNAs around this target such that there was ~2kb of complex sequence flanking the repeat on either side. This way, the aligner should theoretically be able to map an expansion-containing repeat to the correct locus, albeit not necessarily to both sides of the repeat in a single alignment. Next, to avoid losing any sequence information, we set the parameters of Minimap2 to prevent hard clipping with the -Y option. Then, we could clearly observe that most of the reads from expanded alleles would align upstream of the locus in a primary alignment and downstream in a supplementary alignment (or vice versa). This phenomenon did not interfere with our ability to estimate repeat copy number because we took each read that aligned by even 1 bp to the locus and realigned each read to the flanking sequences up- and downstream of the repeat using a striped Smith-Waterman method to identify spanning reads and estimate repeat copy number. These methods are clarified with new language in “**Repeat copy number estimation in ALS samples” section of the Methods.**

5. *… ReadUntil … has recently been renamed to ReadFish ... and the name should be changed accordingly in the text. Furthermore, it should be stated clearly that only off-target reads are, in principle, rejected (or on-target reads retained).*

We thank the reviewer for bringing this change to our attention. All instances of “ReadUntil” have been changed to “ReadFish”. Likewise, **Line 135** of the introduction has been changed to state: “**The method utilizes real-time sequence identification to allow off-target DNA fragments to be rejected from nanopores**”

6. *… NanoSatellite is not currently maintained … Other tools for tandem repeat profiling which will benefit from improvements of Guppy basecalling performances exist and, among these, TRiCoLOR (*[*https://doi.org/10.1093/gigascience/giaa101*](https://doi.org/10.1093/gigascience/giaa101)*) seem in high-quality.*

We have updated the manuscript to reflect the changes to Nanosatellite identified by Reviewer 1. In this case, we have changed the original verbiage in the discussion referencing Nanosatellite to the following, starting on **Line 382:** **“As the performance of Guppy continues to improve, methods that have been developed to detect tandem repeat in long-read sequencing data will also improve. For example, STRique (19) and TRiCoLoR (48), which detect repeat expansions from aligned reads, have already outpaced Nanosatellite, a repeat detection algorithm designed to circumvent issues with base calling by detecting repeats from raw signal data (41).”**

Reviewer #2

1. *…I have found these references that might be nice to cite here as well: (López-Girona et al., 2020; Watson et al., 2020) and also (Bennett-Baker & Mueller, 2017; Jiang et al., 2015; Jiang & Zhu, 2016; Lee, Larionov, & Kouprina, 2015; Loose, 2018).*

We thank the reviewer for bringing these manuscripts to our attention. We have cited Lopez, Watson, Bennet, and Jiang to the introduction (**Line 123-125**). The method put forth by *Lee, Larionov, & Kouprina, 2015* is discussed in the discussion section (**Line 343**) and provides an interesting example of a specific non-sequencing application where CaBagE could potentially increase efficiency.

2. ***Figure 2A****. It would be nice to add the orientation of the guide RNAs followed by their PAMs at either side of the scissors somehow (maybe a simple arrow will do the job), so it would be clearer what PAM-in and PAM-out orientation means by simply looking the figure.*

We have changed Figure 2A in accordance with the reviewer’s suggestion. We agree that this change clarifies what is meant by PAM-in and PAM-out in this context.

3. *…The discussion about the performance of CaBagE versus nCats in lines 287-293 is a bit vague… comment on the applicability of your method versus nCat. Why people should try CaBagE…*

We agree with the reviewer’s observation that nCATs is cheaper, faster, and more efficient than CaBagE. Therefore, we emphasize in our manuscript the distinguishing feature of CaBagE, which is the physical enrichment of a target locus in the DNA sample. As such, we have expanded our discussion of the potential applications of PCR-free physical target enrichment of a DNA sample beyond sequencing (**Lines 330-346**). These include molecular methods such as PCR-free cloning and Southern blot. During the time that we were developing CaBagE, many other methods were proposed (as Reviewer 2 aptly points out). Despite CaBagE not achieving superior performance, we feel that it is important to present as an alternative with distinguishing features that may prove useful.

4. *…by CaBagE you restrict the coverage to those un-fragmented and perfectly Cas9-digested DNA molecules*… *By removing the single side sequencing reads you also restrict the use of the CRISPR-Cas9 enrichment methodology to other further applications.*

This is an astute observation. While not much can be done in terms of the method itself, we have added a sentence to the discussion to ensure that this nuance is clear to all readers. **Line 318: “There is also an increased sensitivity of CaBagE to fragmentation between Cas9 binding sites, where any break in DNA or failure of binding by either of the guides will result in degradation of the target molecule.”**

5. *An interesting point to highlight at discussion section would be to add a table including price and time comparison between doing nCats and CaBagE.*

As mentioned above, there is no cost or time advantage to CaBagE compared to nCATs. In order to make this clear to the readership, we included the following on **Line 315** of the discussion **“Specifically, CaBagE costs approximately $9.40 more per run than nCATs and requires 2 additional hours of hands-off incubation time.”**

6. *…the section ‘repeat copy number estimation’ on discussion around line 386 needs some attention. The long paragraph starting by ‘Next, spanning reads were realigned…’ needs to be broken in different sentences for clarification or be explained in a simpler way as it gets confusing at the second line.*

This section has been rewritten and broken up into 3 paragraphs for clarity (**Lines 462-477**)

7. *Why do you use* *sequencing buffer (SQB) with water and flushing buffer (FB) for priming the pore rather than using FB with sequencing tether? This is a modification from the usual protocol. Do you see any improvement in the library run by doing this? This will be nice to comment in the discussion…*

We adapted this protocol following a personal communication with Timothy Gilpatrick, lead author of the nCATs method. He and his team were instructed to modify the protocol by affiliates at Oxford Nanopore. Both he and our group obtained significantly higher yields using this strategy as compared to the standard protocol. I have added a clarifying line to the methods section **(Line 443) “Flow cells with >800 active pores following Platform QC were primed according to the adapted protocol from Gilpatrick et al (24) with 800μL of Flush Buffer followed by a second priming with priming mix (70μL Sequencing Buffer + 70μL nuclease-free water + 70μL Flush Buffer).”** However, the current version of the amplification-free targeted sequencing kit currently available from Oxford Nanopore no longer employs this strategy.

8. *You should provide a Github repository link (or similar service, bitbucket, source forge, gitlab…) containing the analysis code used in this study as well as provide access to the raw and sequencing data generated.*

We have created a Github repository (<https://github.com/adw222/CaBagE-manuscript>) that contains analysis code for preprocessing and repeat copy number estimation. We have provided a link in the manuscript on **Line 479.** We have also included the complete and detailed wet lab protocol on the Github repo Wiki. Fastq files that were generated from healthy DNA donors are also contained there. Sequencing data generated from *C9orf72* repeat expansion carriers will be made available in dbGap in accordance with NINDS requirements.

We thank the referees again for their detailed review and for providing us with a constructive feedback on our work. Incorporation of these comments has strengthened and clarified the manuscript.
